# Is Inhaled Colostrum as Effective as Inhaled Lavender Essential Oil for Pain Control in Neonatal Frenotomies? A Prospective, Randomized Clinical Trial

**DOI:** 10.3390/children12080982

**Published:** 2025-07-26

**Authors:** Silvia Maya-Enero, Júlia Candel-Pau, Beatriz Valle-Del Barrio, Montserrat Fàbregas-Mitjans, Sandra Prieto-Paja, María Ángeles López-Vílchez

**Affiliations:** Department of Neonatology, Service of Pediatrics, Hospital del Mar, Parc de Salut Mar, Universitat Pompeu Fabra, Passeig Marítim 25-29, 08003 Barcelona, Spain; jcandelpau@hmar.cat (J.C.-P.); bvalle@hmar.cat (B.V.-D.B.); mfabregas@hmar.cat (M.F.-M.); sprieto@hmar.cat (S.P.-P.); malopez@hmar.cat (M.Á.L.-V.)

**Keywords:** ankyloglossia, aromatherapy, colostrum, frenotomy, lavender essential oil

## Abstract

Background/Objectives: Neonatal pain must be treated due to its potential short- and long-term adverse effects. A frenotomy is a painful procedure where common strategies to relieve pain (oral sucrose solutions and sucking) cannot be used because the technique is performed on the tongue. Lavender essential oil (LEO) is useful in treating pain during blood sampling, heel punctures, vaccination, and frenotomies. We aimed to determine whether smelling colostrum had similar effects as inhaled LEO during frenotomies. Methods: A prospective, randomized clinical trial was carried out with neonates who underwent a frenotomy for ankyloglossia between September 2023 and June 2024. We assessed pain using the NIPS score, heart rate, oxygen saturation, and crying time. After obtaining parental informed consent, we randomized patients into experimental and control groups. In both groups, we performed swaddling, administered 1 mL of oral sucrose, and let the newborn suck for 2 min. In the experimental group, we placed a gauze pad with two drops of colostrum, whereas in the control group, we used one drop of LEO 2 cm under the neonate’s nose prior to and during the frenotomy. Results: We enrolled 142 patients (71 experimental cases and 71 controls). The experimental group showed lower crying times (28.0 vs. 40.2 s, *p* = 0.03). Both groups showed similar NIPS scores (1.4 vs. 1.5, *p* = 0.28). We observed no side effects in either of the groups. Conclusions: Inhaled colostrum and LEO help relieve pain in neonates who undergo a frenotomy for ankyloglossia and have no side effects. Aromatherapy with colostrum may decrease crying time during the frenotomy.

## 1. Introduction

Neonates routinely undergo painful procedures, such as blood glucose level control or blood sampling, for the early diagnosis of inborn errors of metabolism. Over the last few years, neonatal units have shown an increasing interest in studying the pain that newborns feel. Historically, newborns were thought to have no pain due to the immaturity of their nervous system [1,2]. Evidence, however, shows that newborns feel pain and may even have increased sensitivity to it and to its long-term negative effects due to this immaturity [3,4], and that repeated unrelieved pain can cause adverse physiologic effects, such as hemodynamic instability, decreased oxygen saturation, and increased intracranial pressure in the short term, and affect neurodevelopment in the long term [3,5,6]. However, appropriate analgesia is not always provided [6,7,8]. Development-based care focuses on avoiding painful procedures as much as possible, grouping interventions in order to minimally manipulate the newborn, and managing pain, either by administering analgesia, swaddling the patient, or combining the two [3,4,6,7,9,10,11,12,13]. Non-pharmacological pain relief is important in neonatology due to the potential adverse effects of pharmacological treatments. It includes sensory stimulation (positioning or swaddling, vestibular action or rocking, aromatherapy, non-nutritive sucking, and musical therapy), nutritive (oral sweet solutions), and maternal interventions (maternal odor and voice, breastfeeding, and skin-to-skin contact) [3,4,6,9,14,15,16,17,18,19,20,21,22,23,24,25,26,27,28,29]. Oral sucrose solutions reduce the signs of pain but do not eliminate it, and this is why they should be used in combination with other non-pharmacological interventions [6,11,13,19,26,27,30,31].

In our hospital, we perform painful techniques following the administration of oral sucrose, swaddling, and allowing neonates to breastfeed or suck, which helps to prevent crying. However, such actions are not enough during frenotomies because patients cannot suck during the procedure. In a previous clinical trial, we observed that signs of pain decreased when using inhaled lavender essential oil (LEO) (*Lavandula angustifolia* ssp. *angustifolia*) while performing the frenotomies. Thus, we introduced inhaled LEO in our routine practice [32]. We also performed a clinical trial comparing the use of inhaled LEO and vanilla essential oil and found no differences between them [33].

It is known that colostrum, either inhaled or ingested, decreases pain in neonates and infants. Colostrum is a familiar scent, and there are several studies that found that a familiar scent may have a calming effect on neonates subjected to a painful procedure [34,35,36]. Although the exact mechanism of colostrum’s pain-relieving effect is unknown, proximity to the mother and skin contact may mediate the release of beta endorphins and oxytocin, as previously described by McNair et al. and Hofer et al. [37,38]. There are studies that demonstrate that both colostrum and LEO are effective for neonatal pain relief during blood sampling, frenotomies, or endotracheal suctioning, among others [11,28,32,39,40]. Nevertheless, as far as we know, no previous studies have analyzed the analgesic effect of inhaled colostrum during the frenotomy nor directly compared its analgesic effect with that of inhaled LEO to relieve pain in neonatal frenotomies. The aim of this study was to determine whether the use of inhaled colostrum was as effective as the use of inhaled LEO in reducing pain during frenotomies in healthy, full-term neonates.

## 2. Materials and Methods

We conducted a prospective, randomized clinical trial (ClinicalTrials.gov ID: NCT06869902). Our hospital’s Ethics Committee (CEIm-PSMAR) approved this study (reference code: 2023/10996). Prior to patient enrollment, we obtained signed informed consent from the neonate’s parents. This study was conducted according to the ethics code of the Barcelona Medical Association and the principles of the Helsinki–Fortaleza Declaration of 2013 at the neonatal unit of a tertiary care hospital in Barcelona, Spain, within an area of influence of approximately 400,000 people, which experiences approximately 1400 births per year. The target population for this study, and, thus, the inclusion criteria, were healthy full-term neonates born at our center or who were younger than 15 days old and referred for a frenotomy with ankyloglossia according to the Hazelbaker tool [41] (Appendix A) between September 2023 and June 2024. We assessed for the presence of ankyloglossia as part of the routine neonatal evaluation, using the Hazelbaker tool to evaluate its impact on tongue movement and breastfeeding [41]. According to the Hazelbaker tool (see Appendix A), ankyloglossia exists if appearance scores 8 points or less and/or function scores 11 points or less. We offer a frenotomy to all patients with ankyloglossia. During the study period, if we identified a patient with ankyloglossia, we offered the patient’s parents the opportunity to participate in this study.

Patients were enrolled if their parents agreed and signed written informed consent. We only included patients who were breastfed, as we may need to obtain colostrum after randomization. Enrolled patients were randomized into the experimental or control groups through simple random sampling using sequentially numbered containers. During the frenotomy, the neonate was taken to the neonatal unit and monitored with a pulse oximeter (COVIDIEN Nellcor Portable SpO2 Patient Monitoring System PM10N, Covidien Ireland Limited, IDA Business & Technology Park, Tullamore, Ireland) before, during, and after the procedure. For both groups, we swaddled, administered 1 mL of oral sucrose, and let the newborn suck for 2 min prior to the procedure. The control group had a 7 cm × 7 cm gauze pad with 1 drop (43.75 mg) of 100% pure LEO (Pranarôm España S.L., Barcelona, Spain) placed 2 cm under their nose for 2 min prior to starting the frenotomy and for the duration of the procedure, whereas the experimental group had a gauze with 2 drops of colostrum from the patient’s mother placed in the same way. We used two drops of colostrum but only one of LEO because the odor of colostrum is more subtle than that of LEO, and in our previous studies [32,33], we had used one drop of LEO, and that is how we routinely perform frenotomies. When a patient was randomized to the experimental group, minutes before performing the frenotomy, we provided his/her mother with gauze so that she could express two drops of colostrum directly on it after washing her hands with water and soap. Once the procedure was completed, we removed the gauze pad and recorded vital signs, whether the baby cried or not, the duration of crying (in seconds), and the Neonatal Infant Pain Scale (NIPS) score on a data collection sheet. If a neonate cried, calming techniques such as holding, swaddling, and sucking were employed. A blinded observer assessed pain in real time by means of crying duration, the NIPS (Appendix B) [42], and whether there was a change in heart rate (HR) and/or oxygen saturation (satO2) before and after the procedure.

In a previously published study where we compared performing frenotomies using complementary analgesia with inhaled LEO alone, we observed a mean (SD) crying time of 14.8 vs. 24.6 (10.8 vs. 27.6) seconds in favor of LEO [32]. In order to detect a difference of 10 s in crying time, we calculated that we needed a sample size of 71 patients per group in order to draw conclusions with a CI of 95% and a power of 80%. Our primary outcome was crying time. We chose to evaluate pain by means of crying time rather than via the NIPS score because, in our previous research (using swaddling and oral sucrose with or without aromatherapy with inhaled LEO or inhaled vanilla essential oil), we obtained NIPS scores of 1.88–2.92 and 2.02–2.38, where a NIPS score of less than 3 indicates no pain. We had observed that neonates had low NIPS scores when inhaling LEO during the frenotomy. However, we did not know if they would exhibit pain when smelling colostrum, and this is the reason why we also assessed the NIPS score in this study. As secondary outcomes, we compared the other variables that we analyzed (NIPS score, HR, and satO2).

We recorded demographic (sex, gestational age, birth weight, and age in hours at the time of the frenotomy) and clinical variables (HR and satO2 before, during, and after the procedure; whether the patient cried or not during the procedure; length of crying time in seconds; the presence of side effects during the procedure (apnea, desaturation, and others); and the highest NIPS score within the first 5 min after the procedure). The attending staff were trained to assess the NIPS score before we started recruiting patients. Even though the patients were swaddled, it was feasible to feel if they moved their legs or arms. The independent variable was the use of inhaled colostrum or inhaled LEO during the frenotomy. The dependent variables were HR and satO2 pre- and post-procedure, the presence of crying and its duration, hours of life at the time of the frenotomy, and the NIPS score. The controlled variables were gestational age, sex, and birth weight.

Statistical analysis: Quantitative variables (gestational age, birth weight, age at frenotomy, HR pre- and post-procedure, increase in HR post-procedure, satO2 pre- and post-procedure, decrease in satO2 post-procedure, and duration of crying) are described using the mean, standard deviation, and range; experimental vs. control groups were compared with a Student’s t-test. We assessed the equality of variances by using Levene’s test and applied the result of the Student’s t-test accordingly. Sex, the presence of crying, and adverse effects between the two groups are presented in percentages and compared using Fisher’s exact test. Statistical significance was set for a *p*-value < 0.05. To perform statistical analyses, we used STATA version 16.1 (StataCorp, College Station, TX, USA).

## 3. Results

We enrolled 142 patients (71 cases and 71 controls) from a total of 194 potential candidates between September 18, 2023 and June 14, 2024. Fifty-two candidates were excluded: 28 parents refused to participate in the study; there was a language barrier with 14 parents; in six cases, the workload did not allow us to have enough personnel available for the study; two patients were not invited to participate because a frenotomy had been performed before; one had received acetaminophen fifteen minutes prior to the procedure; and one patient was isolated due to maternal scabies. The excluded candidates were homogeneous with the included candidates in terms of sex (70.6% male), gestational age (39^4/7^ (1^3/7^) weeks), birth weight (3322.3 (446.9) g), and hours of life (62.5 (66.2) hours). We included 84 male (59.2%) and 58 female (40.8%) newborns. Globally, the mean (SD) gestational age was 39^5/7^ (1^2/7^) weeks, and the mean (SD) birth weight was 3325.7 (467.0) g. The mean (SD) age at the time of the procedure was 66.0 (77.7) hours. Table 1 shows the demographic characteristics of both groups. There were no differences between the two groups in terms of sex, birth weight, gestational age, or age at the time of the frenotomy.

The mean (SD) HR pre-procedure was 130.7 (17.7) bpm, and post-procedure, it was 163.2 (20.4) bpm. The mean (SD) HR increase was 32.5 (19.9) bpm. The mean (SD) SatO2 pre-procedure was 99.4 (1.1) %, and post-procedure, it was 95.3 (4.2) %. The mean (SD) SatO2 decrease was *−*4.0 (4.1) %. A total of 141 patients cried (99.3%), with a mean (SD) crying time of 34.1 (33.2) seconds. All the patients but one (in the LEO group) cried (100% vs. 98.6%). The mean (SD) NIPS score was 1.5 (0.8). There were no differences between the two groups in terms of baseline HR and satO2. Table 2 presents the outcomes of the colostrum (or experimental) group and the control group.

The experimental group showed a minimally higher, non-significant HR increase (33.9 vs. 31.0 s), a lower, non-significant satO2 decrease (*−*3.6 vs. *−*4.5%), shorter crying times (28.0 vs. 40.2 s), and slightly lower, non-significant NIPS scores (1.4 vs. 1.5). Almost all the patients cried in both groups, although neonates in the experimental group cried, on average, 12.2 *s* less compared to the control group. We observed no adverse effects with the use of LEO or colostrum in any of the patients.

## 4. Discussion

To the best of our knowledge, this is the first study to compare the effects of inhaled colostrum vs. inhaled LEO on pain relief during neonatal frenotomies. Aromatherapy uses the healing effects of volatile essential oils in different ways [12,43] and has been widely used for centuries in traditional and modern medicine as a complementary therapy [12,43,44]. Goubet conducted the first study on aromatherapy with neonates in 2003 [34], and, since then, several authors have shown the beneficial effects of aromatherapy on infants [11,12,15,32,33,34,43,45,46,47]. The main aromas used in neonatology are lavender, vanilla, and breast milk [11,14,15,23,24,28,32,33,36,39,40,46,48,49].

The mechanism through which LEO can be used to achieve analgesia is uncertain [11]. It has been inhaled to decrease pain from blood sampling and heel-lances in neonates, neonatal frenotomies, and vaccination at the age of two months [3,11,15,16,33,39,46,50]. Other authors studied the pain relief effect of vanilla essential oil on neonates when performing arterial punctures or frenotomies [33,48]. In a previous study, we compared the complementary analgesic effect of inhaled vanilla vs. inhaled LEO and found no differences between them, for which we continued to use inhaled LEO in our routine practice [33]. Goubet observed that a vanilla scent decreased the crying associated with heel-lances when the neonates had been previously exposed to it, whereas it did not when it was an unfamiliar scent [14]. Furthermore, several authors have studied the non-pharmacologic analgesic effect of colostrum on neonates [28,39,40]. Different studies have demonstrated that neonates calm down when smelling familiar odors, such as their mothers’ breast milk [11,14,48]. Familiar scents are capable of decreasing crying and oxygen consumption in neonates, whereas the same odors have no beneficial effects in newborns who have not been previously exposed to them [35,50]. In our study, the neonates in the colostrum group cried for a shorter time than those in the LEO group. All the neonates in our study were breastfed, for which colostrum was probably a familiar scent for them. However, we did not assess how many times they had been breastfed prior to the frenotomy. Moreover, some of them were as young as five hours old when the frenotomy was performed. Thus, we cannot conclude whether the analgesic effect of colostrum was due to it being a familiar scent for them or not. Several authors have studied the beneficial effects of oral or inhaled colostrum while performing painful techniques, such as endotracheal suctioning on preterm neonates, venipunctures, or heel-lances [27,28,29,35,36,39,40,49,51,52]. We did not administer the colostrum orally but instead allowed the neonates to smell its odor during the frenotomy. Other authors have studied the effects of the smell, not the taste, of colostrum, as we did. As far as we know, no previous studies have used colostrum as a method of aromatherapy during frenotomies.

None of the prior aromatherapy studies performed on infants have described any side effects, including nausea, vomiting, or chills [32,33,48,53,54]. In line with these, we have also not observed any side effects from its use. Therefore, we conclude that using both colostrum and inhaled LEO is safe during a frenotomy. We would like to highlight the fact that even though crying time decreased significantly in the colostrum group (28.0 vs. 40.2 s), a 12 s decrease in crying time may not be clinically significant. Moreover, the large SD that we observed regarding crying time suggests high variability; therefore, we measured the size effect with Glass’s delta test (0.26, 95% CI −0.07; 0.59). This low value and the fact that the measure is not significant suggest that it is clinically impossible to differentiate the population of neonates treated with LEO from those who inhaled colostrum. In addition to this, we observed no differences in NIPS scores between the two groups, even though one patient in the colostrum group had a NIPS score of 5, which indicates severe pain, although she only cried for 20 s. Taking these findings into account, we opted not to change our study protocol, and we continued to perform the frenotomies using inhaled LEO, which is more easily and quickly available. We would have considered a fifty-percent reduction in crying time to be clinically important.

We would like to highlight the fact that we observed longer crying times when using inhaled LEO in this study than in our previous ones (40.2 s in the present study vs. 14.8 s when comparing inhaled LEO with oral sucrose, sucking, and swaddling, and 15.3 s when comparing LEO with vanilla essential oil). There were no differences in these three studies in terms of how the procedure was performed or who performed it. None of the neonates in this study or in our previous studies had been previously exposed to the scent of LEO, which was an unfamiliar scent for all of them. The populations in our three studies were similar in terms of sex (59.2% males in the current study, 52.5% in the study where we compared lavender with oral sucrose, sucking, and swaddling, and 54.2% in the study where we compared LEO vs. vanilla essential oil), gestational age (39^5^, 39^4^, and 39^6^ weeks, respectively), and birth weight (3322, 3307, and 3328 g, respectively). However, age at the time of the frenotomy was higher in the present study (62.5, 47.8, and 43.0 h of life, respectively). In this study, the average age was 72.3 h for the colostrum group and 59.8 h for the LEO group. We hypothesize that a possible explanation for the longer crying times could be the fact that older neonates are more irritable. It is known that neonates are often sleepier and more lethargic in the first 24 to 48 h after birth, and more awake afterward. Even though the age at frenotomy was not statistically different in our study groups, patients in the experimental group were 12.5 h older, meaning that colostrum could be a familiar scent for them, whereas patients in the LEO group were younger. The fact that they were, on average, 10.2 and 17.4 h older than in our previous studies (LEO vs. oral sucrose, sucking, and swaddling, and LEO vs. vanilla essential oil, respectively) could justify the differences in crying time that we found. Indeed, we observed a weak correlation between the variables “crying length” and “hours of life” (r = 0.23, 95%CI 0.7–0.38), which could support our hypothesis. The main difference between this study and our previous two is that the present study was conducted at our new maternity ward, where not all the attending nurses were neonatal nurses, for which swaddling and comfort measures could have been less effective (thus, leading to longer crying times) than in the previous ones, where all the neonates were handled by neonatal nurses.

We acknowledge that this study has limitations. We used one drop of pure LEO with a standard concentration (43.75 mg) but two drops of colostrum. We chose to use two drops instead of only one because the smell of colostrum is much more subtle than that of LEO. However, we did not know the exact concentration of the colostrum, and it could have been heterogeneous among participants. The team that performed the frenotomies was not blinded, because the smell of LEO is too obvious to ignore, but the person who evaluated the study variables was. Even though more than one person performed the frenotomies, the three staff neonatologists who participated in this study have similar experience and training, for which the technique is comparable. We believe that our study is easily reproducible. The NIPS score is composed of multiple behavioral and physiological elements. We assessed the global NIPS score but did not register which particular items were altered in each patient. However, as the global NIPS scores did not differ between the groups, we believe that this analysis would not be clinically significant.

## 5. Conclusions

The use of both colostrum and inhaled LEO helps relieve pain in neonates who undergo a frenotomy for ankyloglossia, with no side effects. Aromatherapy with colostrum may decrease crying time during a frenotomy but not NIPS scores in comparison to inhaled LEO. All the patients but one had NIPS scores that indicated no pain or mild pain. As the differences in crying time were not clinically significant, we opted to continue performing the frenotomies with LEO, given that it has been our standard clinical practice since 2022, when we published the results of our clinical trial. However, we offer inhaled colostrum to families reluctant to use LEO.

## Figures and Tables

**Table 1:** Demographic characteristics of the aromatherapy group and the control group.

	Experimental Group *n* = 71 (%)	Control Group *n* = 71 (%)	*p*-Value
Male newborn	38 (53.5)	46 (64.8)	0.23 ^a^
Female newborn	33 (46.5)	25 (35.2)	
Ratio ♂:♀	2.1:1	2.8:1	
Birth weight (grams) (mean, SD) (range)	3310.4 (478.6)	3341.1 (457.9)	0.69 ^b^
	(2026–4502)	(2138–4106)	
Gestational age (weeks) (mean, SD) (range)	39^5/7^ (1^2/7^)	39^4/7^ (1^2/7^)	0.55 ^b^
	37^1/7^–42^2/7^	37^0/7^–41^6/7^	
Age at frenotomy (hours) (mean, SD) (range)	72.3 (76.8) (5–360)	59.8 (78.8) (5–360)	0.34 ^b^

^a^ Fisher’s exact test, ^b^ Student’s *t*-test.

**Table 2:** Outcomes of the experimental and control groups.

	Experimental Group *n* = 71 (%)	Control Group *n* = 71 (%)	*p*-Value
Heart rate (bpm) pre-procedure (mean, SD) (range) post-procedure (mean, SD) (range)	130.0 (17.2) (93–168) 164.0 (19.6) (84–202)	131.5 (18.3) (88–180) 162.5 (21.3) (20–203)	0.63 ^b^ 0.66 ^b^
Increase in heart rate post-procedure (bpm) (mean, SD) (range)	33.9 (20.4) (−20;+87)	31.0 (19.4) (−18;+80)	0.38 ^b^
Oxygen saturation (%) pre-procedure (mean, SD) (range) post-procedure (mean, SD) (range)	99.5 (1.1) (95–100) 95.8 (3.3) (84–100)	99.3 (1.1) (95–100) 94.8 (4.9) (77–100)	0.46 ^b^ 0.16 ^b^
Decrease in oxygen saturation post-procedure (%) (mean, SD) (range)	−3.6 (3.2) (0;−16)	−4.5 (4.9) (+1;−23)	0.22 ^b^
Crying (seconds) (mean, SD) (range)	28.0 (24.4) (1–150)	40.2 (39.4) (0–182)	0.03 ^b^
Presence of adverse effects (yes, %)	0 (0.0%)	0 (0.0%)	-
NIPS score (mean, SD) (range)	1.4 (0.8) (0–5)	1.5 (0.8) (0–3)	0.28 ^b^

^b^ Student’s *t*-test.

## Data Availability

The data presented in this study are available on request from the corresponding author due to our study data not being in a public repository.

## Abbreviations

The following abbreviations are used in this manuscript:

LEO	Lavender essential oil
NIPS	Neonatal Infant Pain Scale
HR	Heart rate
satO2	Oxygen saturation
SD	Standard deviation
CI	Confidence interval

## Appendix A

**Table A1:** Hazelbaker Assessment Tool for Lingual Frenulum Function (HATLLF).

Appearance Items	Score	Function Items	Score
Appearance of tongue when lifted		Lateralization	
Round or square	2	Complete	2
Slight cleft in tip apparent	1	Body of tongue but not tongue tip	1
Heart-shaped	0	None	0
Elasticity of frenulum		Lift of tongue	
Very elastic (excellent)	2	Tip to mid-mouth	2
Moderately elastic	1	Only edges to mid-mouth	1
Little or no elasticity	0	Tip stays at alveolar ridge or rises to mid-mouth only with jaw closure	0
Length of lingual frenulum when tongue lifted		Extension of tongue	
More than 1 cm or embedded in tongue	2	Tip over lower lip	2
1 cm	1	Tip over lower gum only	1
Less than 1 cm	0	Neither of the above, or anterior or midtongue humps	0
Attachment of lingual frenulum to tongue		Spread of anterior tongue	
Posterior to tip	2	Complete	2
At tip	1	Moderate or partial	1
Notched tip	0	Little or none	0
Attachment of lingual frenulum to inferior alveolar ridge		Cupping	
Attached to the floor of the mouth or well below the ridge	2	Entire edge, firm cup	2
Attached just below the ridge	1	Side edges only, moderate cup	1
Attached at ridge	0	Poor or no cup	0
Total appearance score		Peristalsis	
Function items score ▪ 14: perfect score (regardless of the Appearance item score) ▪ 11: acceptable if the Appearance item score is 10 ▪ <11: function impaired Frenotomy should be considered if management fails. Frenotomy necessary if the Appearance item score is <8.		Complete, anterior to posterior (originates at the tip)	2
		Partial: originating posterior to tip	1
		None or reverse	0
		Snapback	
		None	2
		Periodic	1
		Frequent or with each suck	0
		Total function score	

## Appendix B

**Table A2:** Neonatal Infant Pain Scale. A score greater than 3 indicates pain.

Pain assessment		Score
**Facial expression**		
0-Relaxed muscles	Restful face, neutral expression	
1-Grimace	Tight facial muscles; furrowed brow, chin, jaw (negative facial expression – nose, mouth brow)	
Cry		
0-No cry	Quiet, not crying	
1-Whimper	Mild moaning, intermittent	
2-Vigorous cry	Loud scream; rising, shrill, continuous (Note: silent cry may be scored if baby is intubated as evidenced by obvious mouth and facial movement)	
**Breathing pattern**		
0-Relaxed	Usual pattern for this infant	
1-Change in breathing	Indrawing, irregular, faster than usual; gagging, breath holding	
Arms		
0-Relaxed/restrained	No muscular rigidity; occasional random movements of arms	
1-Flexed/extended	Tense, straight arms; rigid and/or rapid extension, flexion	
Legs		
0-Relaxed/restrained	No muscular rigidity; occasional random movements of legs	
1-Flexed/extended	Tense, straight legs; rigid and/or rapid extension, flexion	
**State of arousal**		
0-Sleeping/awake	Quiet, peaceful, sleeping or alert, random leg movements	
1-Fussy	Alert, restless and thrashing	
